# Supplementary material for: Baseband control of single-electron silicon spin qubits in two dimensions
Source: Nat Commun. 2025 Jul 1;16:5605. doi: 10.1038/s41467-025-60351-x (PMC12218113; doi:10.1038/s41467-025-60351-x)
Supplement: Supplementary file 1 — Supplementary Information [file 41467_2025_60351_MOESM1_ESM.pdf]

Supplementary Information: Baseband control of single-electron silicon spin qubits in two dimensions

Florian K. Unseld,[1,*] Brennan Undseth,[1,*] Eline Raymenants,[1] Yuta Matsumoto,[1]
Sander L. de Snoo,[1] Saurabh Karwal,[2] Oriol Pietx-Casas,[1] Alexander S. Ivlev,[1] Marcel Meyer,[1]
Amir Sammak,[2] Menno Veldhorst,[1] Giordano Scappucci,[1] and Lieven M. K. Vandersypen[1,†]

[1] *QuTech and Kavli Institute of Nanoscience, Delft University of Technology, Lorentzweg 1, 2628 CJ Delft, The Netherlands*
[2] *QuTech and Netherlands Organization for Applied Scientific Research (TNO), Stieltjesweg 1, 2628 CK Delft, Netherlands*

This supplementary information includes:

- Supplementary Notes 1-12
- Supplementary Figures 1-12
- Supplementary Tables I-II
- Supplementary References 1-14

* These authors contributed equally to this work.

† L.M.K.Vandersypen@tudelft.nl

Supplementary Note 1. Deriving spin properties from magnetic field simulations

Here, we review how the stray field from on-chip magnets is calculated and how this field determines the spin physics in a 2D array of quantum dots. This is salient as much of the existing literature focuses on the simplified case of linear spin arrays and fails to predict qubit properties away from the sweet spot at low external magnetic field amplitudes. The EDSR Hamiltonian is useful as it elucidates both how a transverse gradient permits qubit control as well as how electric field fluctuations due to charge noise couple to the qubit. Similar derivations may be found in [1] and [2]. The Hamiltonian has the form:

$$H = -\frac{E_Z}{2}\sigma_z - \frac{\Delta_o}{2}\tau_z + \frac{\Lambda}{2}\tau_x\sigma_x - \frac{\lambda}{2}\tau_x\sigma_z + E(t)\tau_x. \tag{1}$$

σ and τ denote Pauli matrices that act on the 2-level spin state or (truncated) 2-level orbital state respectively. $E_Z = g\mu_\mathrm{B}B_\mathrm{tot}$ is the Zeeman splitting. $g = 2$ is the electron spin g-factor, μ_0 is the Bohr magneton, B_{tot} is the magnitude of the total magnetic field present at the dot, and $\hbar$ is the reduced Planck's constant. We make the simplifying approximation that the g-factor for an electron spin in silicon is isotropic such that the spin's quantization axis is effectively set by the direction of the total magnetic field vector.

Δ_o is the orbital energy splitting of the dot, which is typically on the order of 1 meV. Although some valley-orbit hybridization may take place, for simplicity we assume it is the orbit-like dipole of length $\langle g|\hat{r}|e\rangle = a_0/\sqrt{2}$ that dominates electric coupling to the charge state of the confined electron. $a_0 = \sqrt{\hbar^2/m^*\Delta_0}$ is the length scale of the dot. For the silicon quantum well, m^* is 19% of the free electron mass.

$E(t) = E^\mathrm{drive}(t) + E^\mathrm{noise}(t)$ represents a time-dependent electric field that may consist of an intentional drive as well as environmental charge noise. The electric driving field is given by $E^\mathrm{drive}(t) = eE^\mathrm{drive}_\mathrm{ac}a_0\cos(\omega t)/\sqrt{2}$ and is oriented along the in-plane unit vector $\hat{\mathbf{r}} = r_x\hat{\mathbf{x}} + r_y\hat{\mathbf{y}}$. We assume any out-of-plane component does not participate in the EDSR mechanism. e is the elementary charge of the electron and $E^\mathrm{drive}_\mathrm{ac} \approx 1000\,\mathrm{V/m}$ is the amplitude of the electric field. Similarly, charge noise fluctuations of a particular frequency ω' may be expressed as $E^\mathrm{noise}(t) = eE^\mathrm{noise}_\mathrm{ac}a_0\cos(\omega' t)/\sqrt{2}$. The amplitude of the fluctuations $E^\mathrm{noise}_\mathrm{ac} \approx 10\,\mathrm{V/m}$ may be estimated from charge noise measurements.

$\Lambda = g\mu_\mathrm{B}\left|\frac{dB_\perp}{dr}\right|\frac{a_0}{\sqrt{2}}$ gives the energy scale for the synthetic spin-orbit coupling where $\left|\frac{dB_\perp}{dr}\right|$ is the slanting field perpendicular to the quantization axis calculated along the axis of the time-dependent electric fluctuations. The orientation of $B_\perp$ is ambiguous, because there are two orthogonal axes to the quantization axis. This would give rise to $\tau_x\sigma_x$ and $\tau_x\sigma_y$ couplings in the Hamiltonian. By rotating the spin basis about the quantization axis, these contributions can be combined into a single energy scale. $\lambda = g\mu_\mathrm{B}\left|\frac{dB_\mathrm{tot}}{dr}\right|\frac{a_0}{\sqrt{2}}$ describes the effect of the decoherence gradient, whereby the qubit energy may fluctuate due to charge noise. While we can engineer the orientation of an applied electric drive, charge noise may push the confined electron in any x-y direction, so we aim to calculate a reasonable upper bound of $\left|\frac{dB_\mathrm{tot}}{dr}\right|$.

Considering the spin-orbit coupling and time-dependent fluctuation energy scales as small with respect to the orbital splitting, Schrieffer-Wolff perturbation theory may be used to derive the relevant off-diagonal element Ω for driving spin transitions as well as the diagonal element $\delta\omega_0$ coupling electric fluctuations to the effective qubit energy splitting to first order:

$$\Omega = \frac{g\mu_\mathrm{B}a_0^2\left|\frac{dB_\perp}{dr}\right|eE^\mathrm{drive}_\mathrm{ac}}{2\hbar\Delta_o}, \tag{2}$$

$$\delta\omega_0 = \frac{g\mu_\mathrm{B}e\hbar\left|\frac{dB_\mathrm{tot}}{dr}\right|E^\mathrm{noise}_\mathrm{ac}}{m^*\Delta_o^2}. \tag{3}$$

The synthetic spin-orbit coupling also introduces a small, constant renormalization to the Zeeman splitting, but this is unimportant in the context of practical qubit calibration as it is the hybridized qubit frequency ω_0 which is measured directly. These relations directly imply the oft-stated conditions that good EDSR control ($f_\mathrm{Rabi} = \Omega/2\pi > 5\,\mathrm{MHz}$) is achieved when $\left|\frac{dB_\perp}{dr}\right| > 1\,\mathrm{mT/nm}$ and good charge-noise-limited coherence properties ($T_2^* \approx 1/\delta\omega_0 > 10\,\mathrm{\mu s}$) should restrict $\left|\frac{dB_\mathrm{tot}}{dr}\right| < 0.1\,\mathrm{mT/nm}$. In summary, the total magnetic field, transverse gradient, and decoherence gradient form the most relevant quantities to calculate to predict EDSR behaviour.

In the case of low-field operation when hopping spins may be used for single-qubit gate control, the tip in quantization axes between adjacent quantum dots becomes an important metric. While valley-orbit hybridization and intrinsic spin-orbit coupling will cause some site-to-site variation in the g-tensor of the electron spins in the silicon quantum

well, the spin quantization axis will predominantly be aligned with the direction of the magnetic field vector at the relevant dot locations [3]. The tip angle can therefore also be predicted from an accurate magnetic field simulation with quantitative accuracy limited by the accuracy of both the magnet modelling and the electrostatic confinement of the dots.

In Sections Supplementary Note 2 and Supplementary Note 3, we summarize the magnetic field simulation strategies employed in this paper. Regardless of how the magnetic vector field is computed, the relevant quantities for understanding spin control can be computed in the same way. We represent the sum of the constant external field and ferromagnetic stray field as a total vector field:

$$\mathbf{B} = \begin{pmatrix} B_x \\ B_y \\ B_z \end{pmatrix}. \tag{4}$$

We assume that the qubit locations are point-like as the field varies slowly over the length scale of the electron wavefunction. The total magnetic field B_{tot} setting the Zeeman energy is:

$$B_{\text{tot}} = \sqrt{B_x^2 + B_y^2 + B_z^2}. \tag{5}$$

With the additional assumption that the g-factor of an electron in the conduction band minimum is isotropic, the quantization axis is set by the direction of the total magnetic field:

$$\hat{\mathbf{u}}_{\text{tot}} = \frac{B_x}{B_{\text{tot}}}\hat{\mathbf{x}} + \frac{B_y}{B_{\text{tot}}}\hat{\mathbf{y}} + \frac{B_z}{B_{\text{tot}}}\hat{\mathbf{z}}. \tag{6}$$

We can find the perpendicular axes using the Gram-Schmidt process, taking $\{\hat{\mathbf{u}}_{\text{tot}}, \hat{\mathbf{x}}, \hat{\mathbf{z}}\}$ as the starting set of normal vectors:

$$\hat{\mathbf{u}}_{\perp,1} = \hat{\mathbf{x}} - (\hat{\mathbf{u}}_{\text{tot}} \cdot \hat{\mathbf{x}})\hat{\mathbf{u}}_{\text{tot}}, \hat{\mathbf{u}}_{\perp,2} = \hat{\mathbf{z}} - (\hat{\mathbf{u}}_{\text{tot}} \cdot \hat{\mathbf{z}})\hat{\mathbf{u}}_{\text{tot}} - (\hat{\mathbf{u}}_{\perp,1} \cdot \hat{\mathbf{z}})\hat{\mathbf{u}}_{\perp,1}, \tag{7}$$

The transverse fields are therefore $B_{\perp,1/2} = \mathbf{B} \cdot \hat{\mathbf{u}}_{\perp,1/2}$ and equal 0 at the qubit locations. The total transverse gradient is found by taking the total directional derivative of each gradient along the driving axis:

$$\left|\frac{dB_\perp}{dr}\right| = \sqrt{(\hat{\mathbf{r}} \cdot \nabla B_{\perp,1})^2 + (\hat{\mathbf{r}} \cdot \nabla B_{\perp,2})^2} \tag{8}$$

$$= \sqrt{\left(\frac{\partial B_{\perp,1}}{\partial x} r_x + \frac{\partial B_{\perp,1}}{\partial y} r_y\right)^2 + \left(\frac{\partial B_{\perp,2}}{\partial x} r_x + \frac{\partial B_{\perp,2}}{\partial y} r_y\right)^2}. \tag{9}$$

We refer to Supplementary Eq. (9) as the driving gradient.

For a particular fluctuation axis $\hat{\mathbf{r}'} = r'_x\hat{\mathbf{x}} + r'_y\hat{\mathbf{y}}$, the decoherence gradient is given by the directional derivative:

$$\left|\frac{dB_{\text{tot}}}{dr}\right| = \left|\hat{\mathbf{r}'} \cdot \nabla B_{\text{tot}}\right|. \tag{10}$$

Since we don't know along which axis charge noise will predominantly push the dot, we can estimate a bound by taking the norm of the in-plane gradient:

$$\left|\frac{dB_{\text{tot}}}{dr}\right|_{\max} \approx \sqrt{(\hat{\mathbf{x}} \cdot \nabla B_{\text{tot}})^2 + (\hat{\mathbf{y}} \cdot \nabla B_{\text{tot}})^2}. \tag{11}$$

We refer to Supplementary Eq. (11) as the decoherence gradient.

Often, the driving axis is designed to align with the same cartesian axis as the external field (e.g. $\hat{\mathbf{y}}$) and the transverse field is dominated by another cartesian component (e.g. $\hat{\mathbf{z}}$) such that the driving gradient can be approximated

as $|\partial B_z/\partial y|$. Similarly, the decoherence gradient can be approximated as $|\partial B_y/\partial x| + |\partial B_y/\partial y|$. However, applying these approximations becomes becomes less accurate in regimes where the inhomogeneous stray field of on-chip magnets dominates the uniform external field.

Finally, the quantization axis tip between adjacent spin sites can be computed from the respective magnetic field vectors $\mathbf{B}_1$ and $\mathbf{B}_2$ at each site as:

$$\theta_{\text{tip}} = \cos^{-1}\left(\frac{\mathbf{B}_1 \cdot \mathbf{B}_2}{B_{1,\text{tot}} B_{2,\text{tot}}}\right) \tag{12}$$

Supplementary Note 2. Micromagnet simulations

For micromagnet simulations where the ferromagnetic material can be approximated as a bulk material, we leverage the efficient magnetic field calculations of the Python package magpylib [4]. Such simulations treat the micromagnet as having a homogeneous polarization with no microscopic crystal structure such that the analytic form of the stray field for constituent magnet shapes can be used to calculate the relevant total magnetic vector field.

There are several sources of uncertainty in such a simulation in our context. At a fundamental level, no time-dynamics or domain wall formation is accounted for in the simulations. See [5] for a detailed discussion of magnet simulation approaches and their limitations. Independent of the simulation method, the accuracy is limited to the level of detail included in the magnet model itself as well as knowledge of the precise location of the accumulated quantum dots.

We therefore aim to extract a useful qualitative picture. First, we model the micromagnet using the nominal design. This excludes roughness due to the underlying gate structure, finite rounding of the magnet edges, and other small misalignments from the fabrication procedure. Second, we use the experimentally measured qubit frequencies to fit the effective homogeneous polarization of the model at a particular external field setting using a least-squares optimization (see Fig. 3). This requires making an assumption about the location of the spins. We take the center coordinate of the plunger gates as the point-like dot location (as indicated by the red dots in the bottom row of Supplementary Fig. 1). A more detailed electrostatic simulation could be leveraged to predict the dot locations with more precision, but it is unclear if this confers any benefit due to the approximate nature of the magnetic simulation itself. With a fitted magnet polarization, a field can be simulated from which relevant parameters may be extracted per Supplementary Note 1.

The trends predicted in terms of addressability, driving gradient and decoherence gradient all qualitatively match our experimental observations. While the model also predicts when quantization axis tips become substantial, a quantitative estimate becomes very sensitive to the estimated dot locations and the microscopic demagnetization of the micromagnets. Leveraging hopping spins through a larger array could be a powerful *in situ* method of characterizing micromagnet behaviour. Direct cryogenic magnetic imaging of patterned micromagnets would be another straightforward means of verifying magnet behavior.

Supplementary Note 3. Nanomagnet simulations

To obtain an accurate picture of the periodic stray fields produced by the nanomagnets in the 2D array (see Supplementary Fig. 2), we use the OOMMF package [7]. We set the nanomagnets' initial magnetization along the y-axis (their longest axis), which we would perform experimentally by applying an external B-field along the y-axis. We employ an energy minimization evolver/driver (MinDriver) to find the energy-relaxed state of the entire 2D array. The relaxed magnetization will be defined by the nanomagnets' material parameters, as well as their geometry (shape anisotropy), pitch and initial magnetization direction. For the results presented in the main text, we choose Fe with material parameters similar to those in [8], i.e. saturation magnetization M_s = 1700 kA/m, exchange stiffness A_{ex} = 21 pJ/m, and 0 K temperature. We aim for a quantum dot pitch of 100 nm, which requires a nanomagnet pitch of about 280 nm in both the vertical and horizontal direction (this is the pitch within the same row or column). To minimize edge effects, we simulate a rather large volume of 3990 nm x 3815 nm x 200 nm with a cell size of 5 nm in all directions. The nanomagnet sizes are 40 nm x 120 nm x 50 nm. The results in the main text present stray fields at a distance of 100 nm below the nanomagnets, mimicking a quantum well positioned 100 nm below the nanomagnet array. Additional simulations suggest that the exact distance is not critical, as favorable conditions for hopping occur at a range of 90 to 140 nm for the specific sizes of the Fe nanomagnets shown here. Moreover, we find that by decreasing the thickness of the Fe nanomagnet to 30 nm, we can decrease the qubit frequency and decoherence gradient further while maintaining the same tip angles. Finally, we note that we are not limited to Fe, as

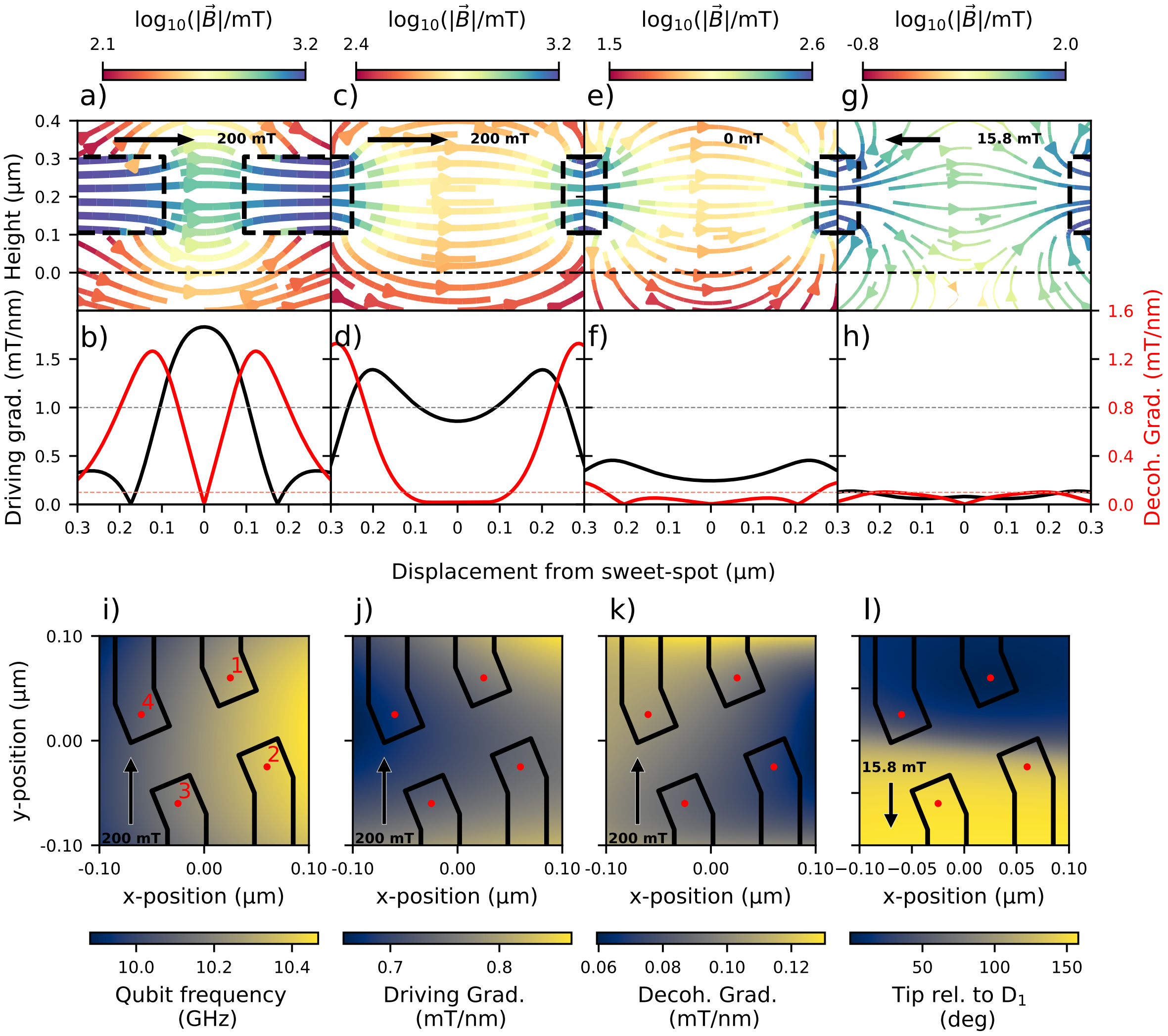

Supplementary Figure 1. **Micromagnet Simulations** a) Magnetic field lines forming in a micromagnet gap of 190 nm as used in [6] when an external field of 200 mT is applied as indicated. No addressability features along the x-axis are included in the magnet design, so the field lines only exist in the y-z plane. The dashed line indicates the level at which the 2DEG and quantum dots would be formed below the magnet. b) The simulated driving gradient (black) along the y-axis and estimated decoherence gradient (red) corresponding to the magnet design in a). The black dashed line indicates the 1 mT/nm minimum threshold for good EDSR control, and the red dashed line indicates the 0.1 mT/nm maximum threshold for good charge-noise resilience. Although a good driving gradient is present, the decoherence sweet spot is only wide enough to support a linear qubit array. c) Magnetic field lines forming in a micromagnet gap of 500 nm similar to the design used in this work when an external field of 200 mT is applied as indicated. d) The simulated driving and decoherence gradients corresponding to the magnet design in c). Good EDSR is generally possible, and the decoherence sweet spot is wider than in a-b). e) Magnetic field lines forming in a micromagnet gap of 500 nm when no external field is applied. f) The simulated driving and decoherence gradients corresponding to the magnet design in e). EDSR is only possible with a stronger driving field, but the sweet spot in which to form good qubits is substantially wider. g) Magnetic field lines forming in a micromagnet gap of 500 nm when an external field of 15.8 mT is applied as indicated. h) The simulated driving and decoherence gradients corresponding to the magnet design in g). EDSR is effectively impossible due to the small gradient, but qubits may be controlled via shuttling. The decoherence sweet spot is still wide. i) Simulated qubit frequencies using the nominal micromagnet design used in this work and an external field of 200 mT. The polarization of the micromagnet used in simulation is inferred based on the measured qubit frequencies. The gate overlay indicates the approximate locations where Qubits 1-4 are formed in the magnetic field. j) The simulated driving gradient along the y-axis with an external field of 200 mT. k) The simulated decoherence gradient with an external field of 200 mT. l) The simulated tip in quantization axis relative to Dot 1 with an external field of 15.8 mT oriented opposite the micromagnet polarization illustrating the substantial tips formed between dots D_1D_2 and D_3D_4.

we further varied ferromagnetic materials and nanomagnet geometries and could obtain similar outcomes. We leave these optimizations for future studies.

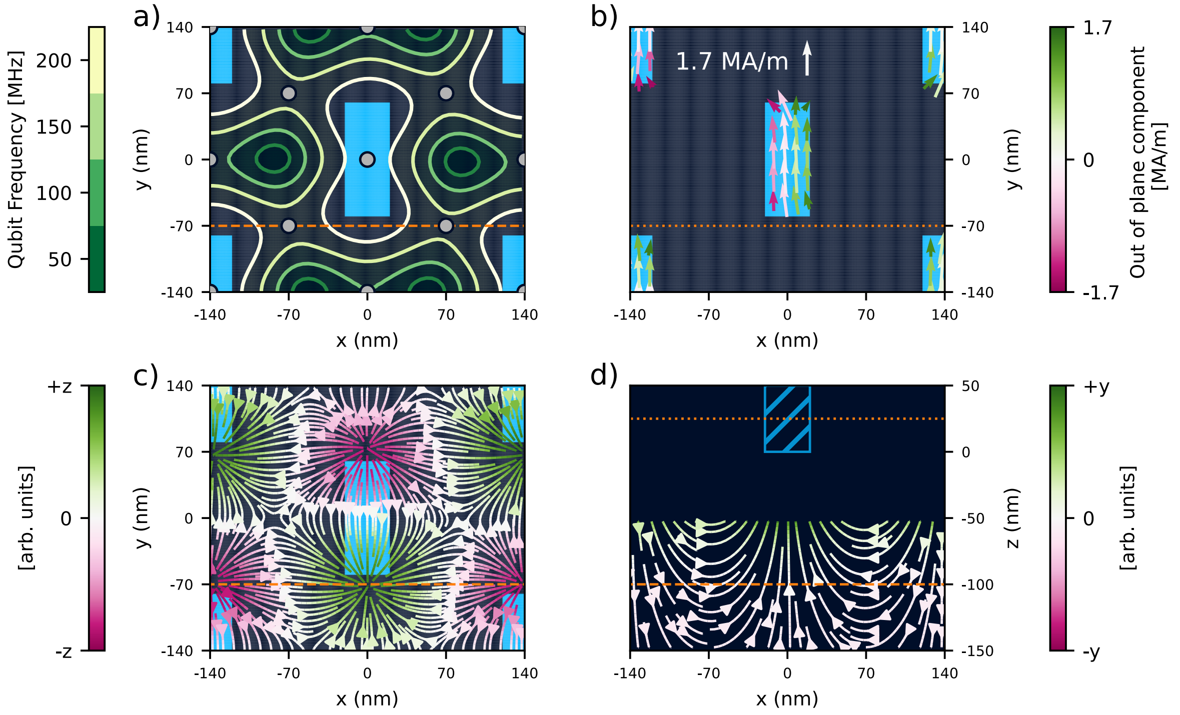

Supplementary Figure 2. **Nanomagnet Simulations** a) Zoomed-in nanomagnet pattern as introduced in Fig. 5. The magnets are shown in bright blue. The bottom surface of the magnets is defined as $z = 0$. For the simulations, we choose Fe as the magnetic material [8] with saturation magnetization $M_s = 1.7\,\mathrm{MA/m}$, exchange stiffness $A_{ex} = 21\,\mathrm{pJ/m^3}$, nanomagnet size $\{l_x, l_y, l_z\} = \{40\,\mathrm{nm}, 120\,\mathrm{nm}, 50\,\mathrm{nm}\}$, cell size $x, y, z = 5\,\mathrm{nm}$ and simulated volume $\{L_x, L_y, L_z\} = \{3990\,\mathrm{nm}, 3815\,\mathrm{nm}, 200\,\mathrm{nm}\}$. The grey dots mark quantum dot locations, and the orange dashed line marks the intersection with the plot shown in panel d). The contour lines show the estimated qubit frequency distribution in a quantum well at $z = -100\,\mathrm{nm}$ assuming a g-factor of 2 for silicon. No external magnetic field is present. b) Magnetization of the nanomagnets at $z = 25\,\mathrm{nm}$. The dotted orange line marks the intersection with the plot shown in panel d). The color of the quiver arrows shows the z-component of the magnetization. The nanomagnets are originally magnetized along the positive y-axis. After the external field is removed, the shape anisotropy maintains the magnetization predominantly in the y-direction. c) Magnetic field lines in the x-y quantum dot plane at $z = -100\,\mathrm{nm}$. The stray field causes the relative quantization axis tips between the dot locations indicated in a) to be about 90 deg. The dashed line indicates the intersection with panel d). d) Stray field lines in the x-z plane at $y = -70\,\mathrm{nm}$. The dashed and dotted orange lines mark the intersections with the planes shown a), b) and c). To maintain good visibility, only the region up to $z = -50\,\mathrm{nm}$ is depicted. The crosshatched blue square indicates the position of the magnetic layer, though there is no nanomagnet present in this plane. The inhomogeneity of the stray field extends relatively far below the magnetic layer allowing ample space for independent gate electrodes.

Supplementary Note 4. Sign extraction from crosstalk experiments

We make use of EDSR to characterize spin physics and encode a set of universal gates with which to perform state tomography. Using the convention $g > 0$ for the electron spin in silicon, the effective two-level Hamiltonian may be given as:

$$H = \omega_0 S_z + 2\Omega \cos(\omega_{\mathrm{MW}} t + \phi) S_x, \tag{13}$$

where $\omega_0 \approx g\mu_0 B_{tot}/\hbar$ is the measured qubit Larmor frequency and Ω is the Rabi frequency due to the effective ac magnetic drive derived in Supplementary Note 1. Here, a microwave drive of frequency ω_{MW} and phase ϕ are used for EDSR. The Loss-DiVincenzo qubit operators are encoded by the spin operators as $\sigma_z = -2S_z/\hbar$ such that $|\downarrow\rangle = |0\rangle$ and $|\uparrow\rangle = |1\rangle$. Therefore, now taking $\hbar = 1$, Supplementary Eq. (13) may be rewritten as:

$$H = -\frac{\omega_0}{2}\sigma_z + \Omega\cos(\omega_{\rm MW}t + \phi)\sigma_x. \tag{14}$$

Experiments at microwave frequencies make use of the rotating frame where $|\tilde{\psi}(t)\rangle = R_z(\omega_{\rm MW}t)\,|\psi(t)\rangle$. In our notation, the unitary operator $R_n(\alpha) = \exp(-i\alpha\hat{n}\cdot\vec{\sigma}/2)$ represents a positive rotation in the Bloch sphere about the unit vector $\hat{n} = (n_x, n_y, n_z)$ by an angle α. The rotating frame Hamiltonian, neglecting fast oscillating terms, is given by:

$$\tilde{H} = R_z(\omega_{\rm MW}t)HR_z^\dagger(\omega_{\rm MW}t) + i\frac{dR_z(\omega_{\rm MW}t)}{dt}R_z^\dagger(\omega_{\rm MW}t) \tag{15}$$

$$= \frac{\Delta}{2}\sigma_z + \frac{\Omega}{2}(\cos\phi\sigma_x - \sin\phi\sigma_y), \tag{16}$$

where $\Delta = \omega_{\rm MW} - \omega_0$. During free evolution in the lab frame for a time t, $H_{\rm free} = -\frac{\omega_0}{2}\sigma_z$, and the state evolves with $U_{\rm free}(t) = R_z(-\omega_0 t)$. During free evolution in the rotating frame, $\tilde{H}_{\rm free} = \frac{\Delta}{2}\sigma_z$, and the state evolves with $\tilde{U}_{\rm free}(t) = R_z(\Delta t)$.

We use parity-mode Pauli spin blockade for measurement and take our observable as $O = (\mathbb{1} - \sigma_z\otimes\sigma_z)/2$ in all cases where $\mathbb{1}$ is the identity operator. We initialize using post-selection into the subspace spanned by $\{|01\rangle, |10\rangle\}$ (see Supplementary Fig.3 for further discussion). Regardless of whether the initial pair is entangled, mixed, or a product state, the following expectation values hold.

We can use a modified Ramsey sequence with a virtual detuning in order to precisely estimate the qubit frequency. After preparation of a superposition state via a gate applied to qubit i, an $R_z^i(\omega_{\rm virtual}t)$ operation is performed via a reference frame update (in the case of resonant control) or a physical wait (in the case of baseband control) prior to measurement:

$$\text{Init} \rightarrow R_x^i(\pi/2) \rightarrow \text{Wait } t \rightarrow R_z^i(\omega_{\rm virtual}t) \rightarrow R_x^i(-\pi/2) \rightarrow \text{Measure } O. \tag{17}$$

The expectation value of the time-ordered sequence is:

$$p_{\rm odd}(t) = A\cos((\Delta + \omega_{\rm virtual})t)\exp(-(t/T_2^*)^a) + B, \tag{18}$$

where $p_{\rm odd}(t)$ is the probability of measuring a state in the $\{|01\rangle, |10\rangle\}$ subspace. A and B are visibility and offset corrections due to constant state preparation and measurement (SPAM) errors, a is a decay constant and T_2^* is the decay time. The frequency of the fitted oscillations $\omega_{\rm fit}^*$ can be related to the Larmor frequency as:

$$\omega_0 = \omega_{\rm MW} - (\omega_{\rm fit}^* - \omega_{\rm virtual}). \tag{19}$$

A similar analysis holds for a Hahn echo pulse sequence:

$$\text{Init} \rightarrow R_x^i(\pi/2) \rightarrow \text{Wait } t/2 \rightarrow R_x^i(\pi) \rightarrow \text{Wait } t/2 \rightarrow R_z^i(\omega_{\rm virtual}t) \rightarrow R_x^i(-\pi/2) \rightarrow \text{Measure } O. \tag{20}$$

The expectation value is given as:

$$p_{\rm odd}(t) = C\cos((\Delta_2 - \Delta_1)/2 + \omega_{\rm virtual})t)\exp(-(t/T_2^{\rm H})^b) + D, \tag{21}$$

where C and D are once again due to SPAM errors, b is a decay constant and T_2^H is the extracted decay time. Here we observe that, contrary to expectation, the free evolution in the rotating frame with frequency $\Delta_1 = \omega_{\rm MW} - \omega_{01}$ before the decoupling pulse may be measurably different than the free evolution with frequency $\Delta_2 = \omega_{\rm MW} - \omega_{02}$ after the pulse. Fitting the decaying oscillations to a frequency $\omega_{\rm fit}^H$ offers information about this systematic difference in Larmor frequency after the echo pulse, as any quasistatic fluctuations are eliminated by the echo pulse:

$$\omega_{01} - \omega_{02} = 2(\omega_{\rm fit}^H - \omega_{\rm virtual}). \tag{22}$$

Supplementary Table I. Comparing the Rabi frequency, T_2^*, $T_2^{\rm Rabi}$, measured X90 gate error $\epsilon_{X90,\rm RB}$, estimated X90 gate error due to quasi-static noise ϵ^*_{X90}, and estimated X90 gate due to decoherence of the dressed qubit $\epsilon^{\rm Rabi}_{X90}$.

Qubit	$f_{\rm Rabi}$ (MHz)	T_2^* (µs)	$T_2^{\rm Rabi}$ (µs)	$\epsilon_{X90,\rm RB}$ (%)	ϵ^*_{X90} (%)	$\epsilon^{\rm Rabi}_{X90}$ (%)
Q1	2.0412(7)	3.31(9)	16(1)	0.33(2)	0.037(2)	0.78(5)
Q2	2.2413(9)	2.02(2)	15(1)	0.20(2)	0.082(2)	0.74(7)
Q3	2.2685(9)	3.57(8)	14(1)	0.61(8)	0.026(1)	0.77(6)
Q4	1.752(1)	2.90(5)	18(2)	0.7(1)	0.065(2)	0.78(8)

An off-resonant pulse may be embedded into the second half of an echo sequence of fixed duration T to probe the transient phase pickup $\theta_t(t_{\rm delay})$ over time $t_{\rm delay}$ as in Fig. 2. In this case, a gate $R_z^i(\theta)$ is swept prior to measurement:

$$\begin{aligned}\text{Init} \rightarrow R_x^i(\pi/2) \rightarrow \text{Wait } T/2 \rightarrow R_x^i(\pi) \rightarrow \text{Wait } T/2 - t_{\rm delay} \rightarrow \text{Burst} \rightarrow \\ \text{Wait } t_{\rm delay} \rightarrow R_z^i(\theta) \rightarrow R_x^i(\pi/2) \rightarrow \text{Measure } O. \quad (23)\end{aligned}$$

The expectation value is given as:

$$p_{\rm odd}(\theta, t_{\rm delay}) = E\cos(T(\Delta_2 - \Delta_1)/2 + \theta_t(t_{\rm delay}) + \theta) + F, \quad (24)$$

where E and F account for the loss of visibility due to the echo decay as well as SPAM errors. As the difference $\Delta_2 - \Delta_1$ is known from the unmodified echo experiment, fitting the oscillation permits extracting θ_t. In the rotating frame, $\theta_t = \int dt_{\rm delay}\Delta(t_{\rm delay})$, and it follows that $\omega_0(t_{\rm delay}) = \omega_{\rm MW} - d\theta_t/dt_{\rm delay}$.

Supplementary Note 5. Estimating the infidelity of resonant qubit control

We may use the measured T_2^* and $T_2^{\rm Rabi}$ timescales to infer which error source contributes more to the resonant single-qubit gate fidelities as measured with randomized benchmarking presented in Figure 2a).

First, we can consider quasi-static frequency fluctuations as captured by the T_2^* coherence timescale. Assuming this is dominated by Gaussian quasi-static noise, the T_2^* relates to the standard deviation σ_f of the qubit frequency fluctuations as $\sigma_f = 1/\sqrt{2}\pi T_2^*$ [9]. Considering the Hamiltonian model for resonant qubit control in Supplementary Eq. (16) with $\phi = 0$, the fidelity of an imperfect unitary gate can be analytically derived in terms of the Rabi frequency $\Omega = 2\pi f_{\rm Rabi}$ and detuning $\Delta \approx \sigma_f$ [10]:

$$F = \frac{d + |\text{Tr}(U^\dagger_{\rm ideal}U_{\rm actual})|^2}{d(d+1)}, \quad (25)$$

where $U_{\rm ideal}$ is the desired unitary operation, $U_{\rm actual}$ is the imperfect operation, and $d = 2$ is the dimension of the single-qubit Hilbert space. In the relevant limit where $\Delta \ll \Omega$, we find the error $\epsilon_{X90} = 1 - F_{X90}$ that originates from quasi-static frequency fluctuations as:

$$\epsilon^*_{X90} \approx \frac{1}{6\pi^2 f^2_{\rm Rabi}(T_2^*)^2}. \quad (26)$$

Similarly, we can consider the error that originates from decoherence while the qubit is subject to the driving field as captured by the $T_2^{\rm Rabi}$ timescale or, equivalently, the quality factor $Q_{1Q} = 2f_{\rm Rabi}T_2^{\rm Rabi}$ conventionally expressed as the number of complete spin flips that fit within the coherence time. Considering exponential decay, the error originating from decoherence while driving is:

$$\epsilon^{\rm Rabi}_{X90} \approx \frac{1}{2Q_{1Q}} = \frac{1}{4f_{\rm Rabi}T_2^{\rm Rabi}}. \quad (27)$$

Table I shows the error estimates from fitting all 4 qubit properties including the measured error $\epsilon_{X90,\rm RB} = 1 - F^{\rm res}_{\rm avg}$ of the primitive X_{90} gate extracted from randomized benchmarking. We can see that the error estimate due to $T_2^{\rm Rabi}$

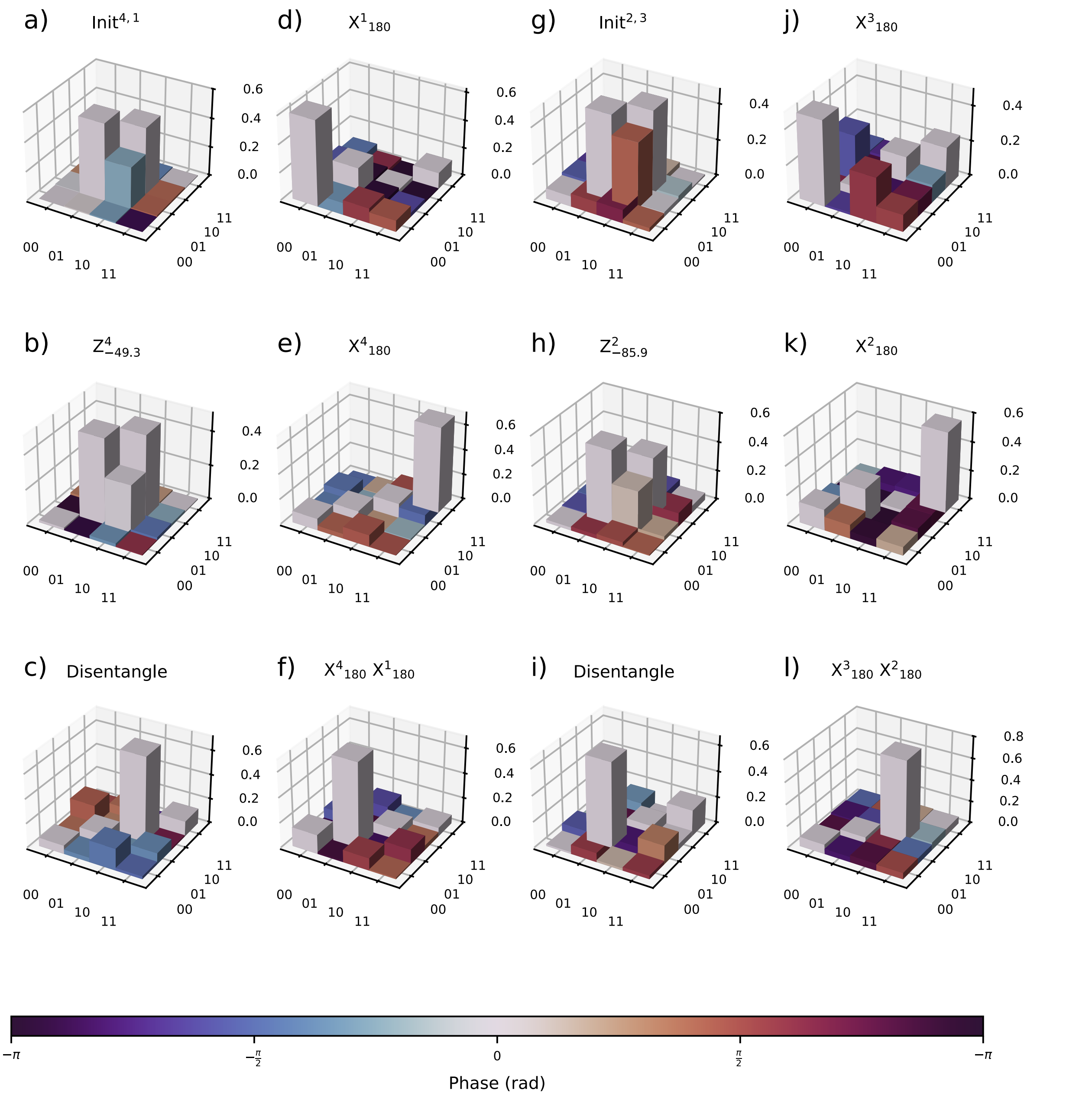

Supplementary Figure 3. **Quantum State Tomography of Qubit Pairs Q4Q1 (a-f) and Q2Q3 (g-l) obtained using EDSR and CZ operations** a)/g) Tomography of the initial state. As we transition quickly out of the (0,2)/(2,0) charge state to the (1,1) charge state, we initialise predominantly in an entangled state in the $\{|01\rangle, |10\rangle\}$ subspace. This diabatic initialization offers more consistent state preparation compared to slow adiabatic ramping. In both cases we still observe a significant classical mixture of the two odd-parity states. For the single-qubit measurement protocols used in this work, a mixed initial state has no impact on the final result. However, such poor initialization has a large impact on most multi-qubit experiments. b)/h) The initialized state after applying a phase operation to produce a $|T_0\rangle$ state. c)/i) The initialized state after disentangling the qubits with a CZ and H gate. d)-f)/j)-l) The disentangled initial state after various X_{180} gates to transform between the product states. A full two-qubit gate set is required to implement quantum state tomography on both Pauli spin blockade pairs illustrating full quantum logic on this device. The poor fidelity of the initial state as well as the poor quality factors of the two-qubit interactions limit the quality of further multi-qubit characterization on this particular device.

decay is about an order of magnitude larger than that originating from quasi-static frequency fluctuations and of the same order as the error measured with randomized benchmarking. We therefore conclude that the T_2^{Rabi} decay dominates the resonant single-qubit gate infidelity.

Lastly, we note that the estimates from Supplementary Eq. (26) and Supplementary Eq. (27) can change meaningfully depending on the exponents of the decay curves and the nature of the microscopic noise. Additionally, the qubit coherence properties were extracted at different points in time than the randomized benchmarking data, so some electrostatic drift could be anticipated. This likely accounts for why there is a discrepancy between the measured and estimated error rates, particularly for Q1 and Q2.

Supplementary Note 6. Hopping spin control and the diabaticity condition

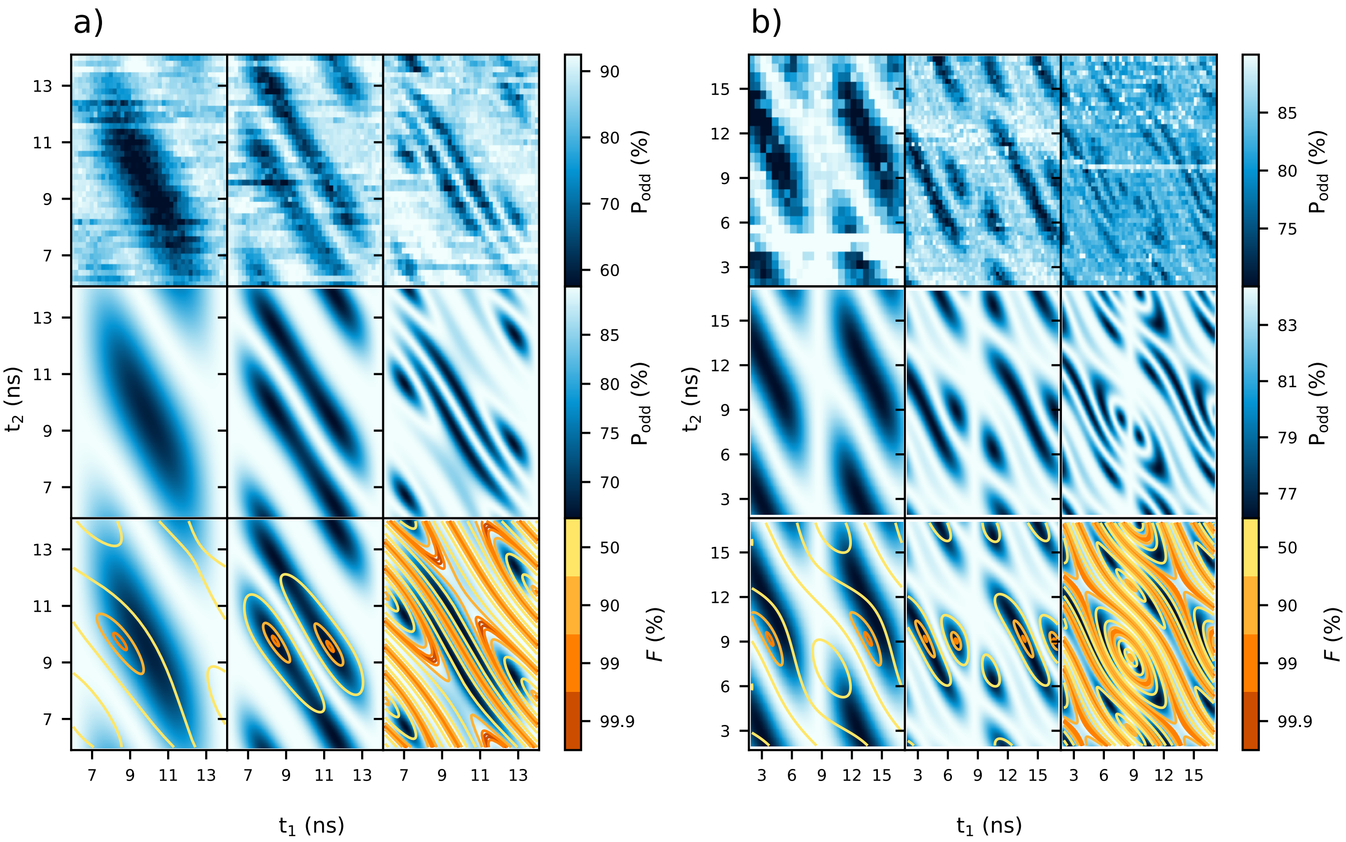

Supplementary Figure 4. **Hopping gate characterisation of D1D2 and D4D3** Raw data (row 1), simulated data (row 2) and estimated gate fidelity (row 3) of one (column 1), two (column 2) and four (column 3) shuttle repetitions for the qubit in D1 (a) and the qubit in D4 (b). In both cases we used two shuttle cycles per repetition. The tunnel coupling for the D_1,D_2 data was fitted to be roughly 18 µeV. For D_4,D_3 the tunnel coupling was not measured. To generate the simulated data sets we fit the four-repetition pattern and extract parameters such as the quantisation axis tip and timing offsets due to finite ramp times. Using the simulated data we calculate the gate fidelity as indicated by the contour plots: for one repetition, we calculate the X_{90} fidelity; for two repetitions, we calculate the X_{180} fidelity; and for four repetitions, we calculate the X_{360} fidelity. The fidelity is calculated by assuming instantaneous shuttling between dots and considering only unitary spin state evolution. In the case of shuttling with Qubit 4, we observe the degradation of the readout signal due to the repeated pulsing on gate P3 (see Methods)

.

Single-spin control via hopping spins can be understood starting from a Hamiltonian describing a single electron in a double quantum dot:

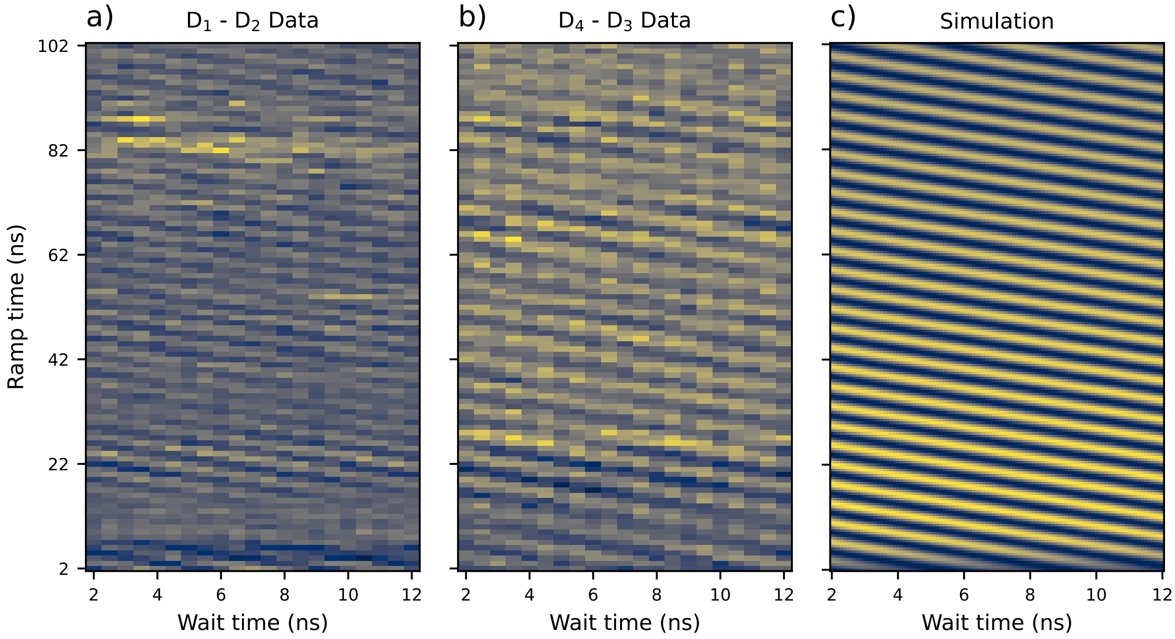

Supplementary Figure 5. **Spin shuttling versus ramp time** Observed spin fraction oscillations when shuttling between a) Dots D_1 and D_2 and b) Dots D_4 and D_3 with a varying ramp time. c) Simulation using parameters extracted from Pair 1-2 in experiment. A total detuning ramp of 300 mV is converted to 10 500 µeV with a combined lever arm of 0.07 meV/mV, and a tunnel coupling of 30 µeV is estimated. The measured qubit frequencies of 240 MHz and 270 MHz are used for the two dot positions along with a measured tip angle of 22 deg. The data in a-b) was collected with an external field of −15 mT. The simulations show that the sudden approximation for spin-state preservation during shuttling is reasonably met in our experiments over a large range of ramp times owing to the very large detuning sweep.

$$
\begin{aligned}
H_{\mathrm{DQD}} &= H_{\mathrm{charge}} + H_{\mathrm{spin}} \\
&= \begin{pmatrix} \epsilon/2 & 0 & t_c & 0 \\ 0 & \epsilon/2 & 0 & t_c \\ t_c & 0 & -\epsilon/2 & 0 \\ 0 & t_c & 0 & -\epsilon/2 \end{pmatrix} + \frac{g\mu_B}{2} \begin{pmatrix} B_{1,\mathrm{tot}} & 0 & 0 & 0 \\ 0 & -B_{1,\mathrm{tot}} & 0 & 0 \\ 0 & 0 & B_{2,\mathrm{tot}}\cos(\theta_{\mathrm{tip}}) & B_{2,\mathrm{tot}}\sin(\theta_{\mathrm{tip}})e^{-i\phi} \\ 0 & 0 & B_{2,\mathrm{tot}}\sin(\theta_{\mathrm{tip}})e^{i\phi} & -B_{2,\mathrm{tot}}\cos(\theta_{\mathrm{tip}}) \end{pmatrix},
\end{aligned} \tag{28}
$$

where ϵ and t_c are the detuning and tunnel coupling describing the double dot system. In the second term, the Zeeman energies at the two sites are considered where θ_{tip} is the polar angle between the two quantization axes and ϕ is the azimuthal angle. This model is directly analogous to the one employed in [11, 12] with the difference that the tip in quantization is attributed to the magnetic field as opposed to the g-tensor. As we are only interested in single-spin physics, we may take $\phi = 0$ with an appropriate choice of coordinate frame.

Fig. 4b) plots the eigenenergies of Supplementary Eq. (28). When the detuning is linearly swept $\epsilon(t) = vt$, the Landau-Zener formula provides a means to estimate the condition for which charge transfer between the two dots is is not adiabatic:

$$
P_{\mathrm{LZ}} = \exp\left(\frac{-2\pi t_c^2}{\hbar v}\right). \tag{29}
$$

Based on a combined vP1+vP2 lever arm of about 0.07 meV/mV and a ramp time of 5 ns, we estimate a detuning ramp speed of about $v = 3000$ µeV/ns. With an estimated tunnel coupling of 40 µeV, we roughly estimate that $0.001 < P_{\mathrm{LZ}} \approx 0.01$. We discuss the implications of this shortly.

Assuming the electron charge is transferred adiabatically, the resulting spin physics is clarified by transforming to the diagonal basis of H_{charge} with a unitary $U = \exp\left(-i\tan^{-1}\left(-2t_c/\epsilon\right)\sigma_y \otimes \sigma_0\right)$ and investigating $H' = UH_{\mathrm{spin}}U^\dagger$ in the sector corresponding to a ground charge state:

$$H' = \frac{g\mu_B}{4}\left[\left(B_{1,\mathrm{tot}}\left(1-\frac{\epsilon}{\sqrt{\epsilon^2+4t_c^2}}\right)+B_{2,\mathrm{tot}}\cos\theta_{\mathrm{tip}}\left(1+\frac{\epsilon}{\sqrt{\epsilon^2+4t_c^2}}\right)\right)\sigma_z + \left(B_{2,\mathrm{tot}}\sin\theta_{\mathrm{tip}}\left(1+\frac{\epsilon}{\sqrt{\epsilon^2+4t_c^2}}\right)\right)\sigma_x\right] \quad (30)$$

Fig. 4c) plots the components of Supplementary Eq. (30). A clear detuning-dependent step arises that is not evident from inspecting the energy level diagram alone. The sudden approximation quantifies how quickly such a Hamiltonian step needs to be traversed in order for the initial quantum state to be preserved:

$$t \ll \frac{2\pi\hbar}{\Delta E}, \quad (31)$$

where $\Delta E \approx 0.1\,\mu\mathrm{eV}$ is the difference between the relevant eigenvalues of H'. This suggests the time interval needs to be less than 1 ns. As the energetic width of the step is about $4t_c$ for the case of our linear detuning ramp, the step is crossed in about 60 ps. In fact, even a total ramp time much longer than was used for the logical gate implementation may be sufficient. This is observed in Supplementary Fig. 5 where ramp times extending to several tens of nanoseconds were used while still observing evidence of sudden spin state transfer between both dot pairs 1-2 and 3-4. Simulations of the time-dependent Schrodinger equation with estimates of experimental parameters corroborate this. We remark that certain ramp times are nontrivially associated with oscillations of different visibility. For example 12 µs ramps consistently resulted in poor visibility despite longer ramps of 22 µs showing higher visibility. The origin of this effect is unknown to us, but it may be related to the microscopic valley-orbit structure of the double-dot system.

The estimated 0.1-1% probability of a diabatic transition at the charge anticrossing is one of the main limitations for the maximum single-qubit gate fidelity that we achieved via hopping spins. This was due to a particularly limited window of device stability within which DC voltages could be tuned to either increase the tunnel coupling or decrease the detuning range necessary to shuttle the electron. Supplementary Eq. (29) and Supplementary Eq. (31) represent a fundamental trade-off, as we desire an adiabatic charge transition with a diabatic spin transition. However, the exponential dependence of the Landau-Zener formula implies that even a modest increase of tunnel coupling greatly enhances the quality of charge transfer while still safely satisfying the sudden approximation. Furthermore, the increased susceptibility to charge noise that occurs when the spin and charge are maximally hybridized means that a fast shuttling time is preferable.

As the micromagnet design and gate layout in the present device was not intended for electron shuttling, there is almost certainly substantial room for improved fidelities by tailoring device design to the conditions for performing shuttling gates. Building upon the understanding of the depolarization of the micromagnet under low-field conditions, engineering quantum axis tips well above 45 degrees between adjacent dots should be easily achievable while keeping qubit frequencies on the order of 100 MHz. Furthermore, being able to modulate the tunnel coupling to the range of 50-100 µeV would substantially improve the quality of adiabatic charge shuttling and should lead to enhanced gate operation.

Supplementary Note 7. Estimating leakage with blind randomized benchmarking

In Figure 6, we present two examples of the blind RB fitting protocol described in the Methods. The two datasets used were calibrated when different Larmor frequencies and tip angles were implemented in the dots, so the two results cannot be compared directly. However, the hopping gate tune-up procedure was the same in both cases. The data in Figure 6a) was taken when the virtual barrier $vB12$ was approximately 100 mV lower than in Figure 6b), which is the same data presented in Figure 4f) of the main text, and we estimate the tunnel coupling between dots to be 18 µeV and 48 µeV in the two cases respectively. The hopping gate suffers from substantially worse leakage when the tunnel coupling is lower, and the blind RB protocol estimates this error to be an order of magnitude lower when the tunnel coupling is raised to the condition where the best hopping gate fidelity was achieved in this device. The Clifford gate fidelity extracted from Figure 6b) is estimated to be 99.52(9)%, which is comparable to the values of 99.01(11)% and 99.49(7)% obtained from standard RB fits for the odd and even parity decay curves respectively.

Blind RB offers limited information about where the state is leaking. In all instances of randomized benchmarking that exhibited signs of leakage, the trend converges towards the odd spin parity signal (i.e. a (2,0) charge state measurement). One explanation consistent with this observation is leakage to excited valley-orbit states. In the discussion in Supplementary Note 6, we did not discuss the role that the valley-orbit state plays in the shuttling

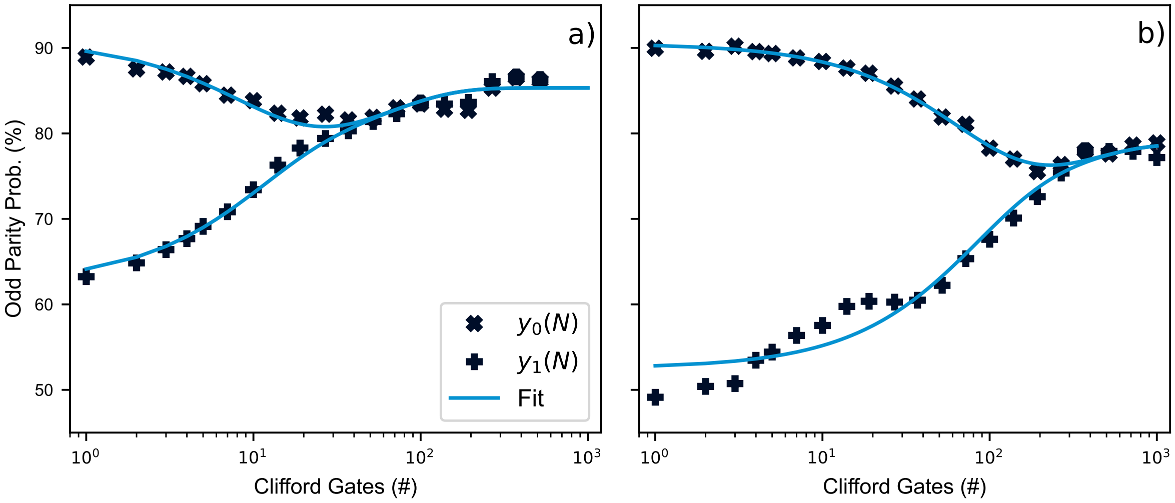

Supplementary Figure 6. **Blind randomized benchmarking** a) Randomized benchmarking performed when the tunnel coupling is estimated to be approximately 18 µeV. Fitting to the blind RB theory results in $A = 0.853$, $B = 0.141$, $C = -0.086$, $p = 0.098$ and $q = 0.017$ and yields a leakage rate of $\Gamma = 1.0(2)\%$, a total error rate of $\epsilon = 5.4(2)\%$, and a qubit error rate of $\epsilon = 4.4(2)\%$ for a Clifford gate fidelity estimate of 94.6(2)%. Each decay curve is the average of 250 random Clifford circuits. (b) Randomized benchmarking performed when the tunnel coupling is estimated to be approximately 48 µeV. Fitting to the blind RB theory results in $A = 0.788$, $B = 0.190$, $C = -0.073$, $p = 0.013$ and $q = 0.003$ and yields a leakage rate of $\Gamma = 0.13(5)\%$, a total error rate of $\epsilon = 0.74(5)\%$, and a qubit error rate of $\epsilon = 0.61(5)\%$ for a Clifford gate fidelity estimate of 99.26(5)%. Each decay curve is the average of 250 random Clifford circuits.

physics. Although diabatic passage through the charge avoided crossing may impede charge transfer, it is also possible that the charge does tunnel between dots but excites into a higher valley-like state. If, at the end of the random Clifford circuit, the charge state is excited to a different valley-orbit state, it is possible that Pauli spin blockade is lifted, resulting in tunneling to the (2,0) charge state during readout.

Supplementary Note 8. Experimental setup

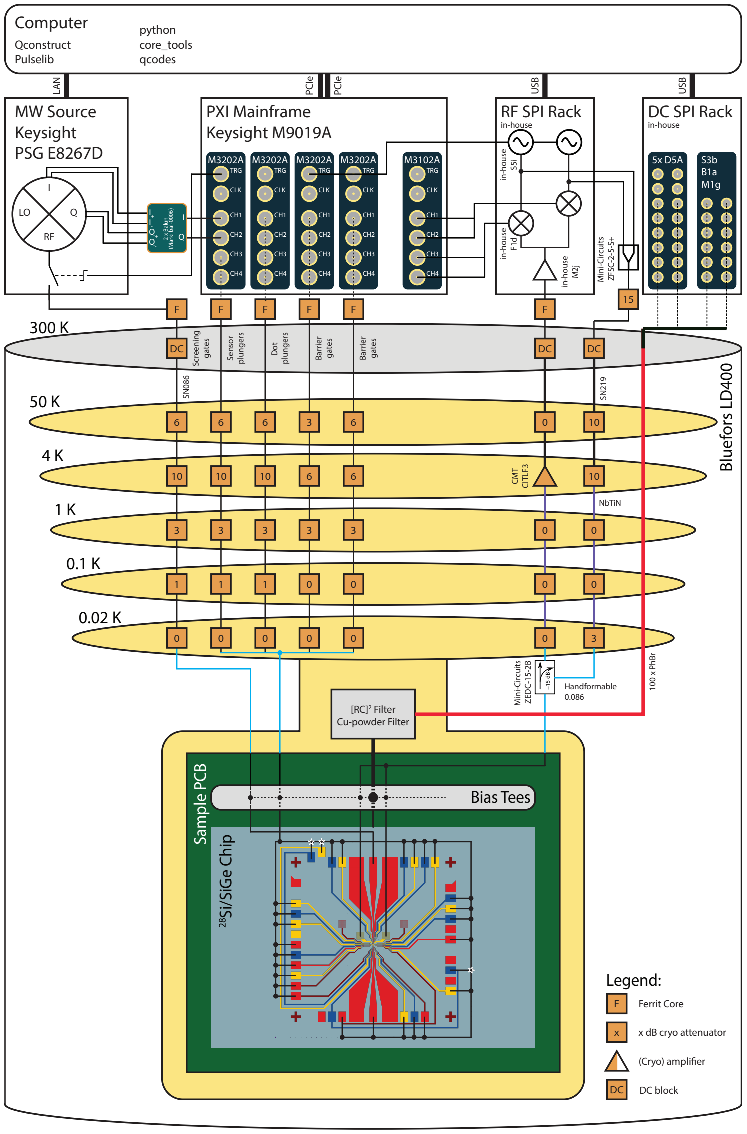

Supplementary Figure 7. **Schematic of the experimental setup** Arbitrary Waveform Generators (AWG, Keysight M3202A) in a PXI chassis are used to generate baseband pulses as well as I/Q input signals for the microwave (MW) vector source (Keysight PSG E8267D). Baseband signals are supplied to all plunger and barrier gates comprising the 2x2 array along with both sensing dot plunger gates. Lines routed to barrier gates are attenuated less to allow for larger voltage pulses on these gates. Wideband modulation with differential I/Q inputs is implemented using Balun's (Marki bal-0006). Ferrite cores are used to reduce low-frequency noise. Double DC blocks (Pasternack PE8210) are used on the microwave drive line and rf-reflectometry readout lines. DC voltages are supplied by battery-powered low-noise voltage sources and are filtered with low-pass RC filters and copper-powder filters before being combined on the sample PCB using bias tees with RC time constants of 100 ms. Two carrier frequencies are generated using homemade sources that are triggered by AWG marker channels and routed via bias tees to two readout tank circuits which are connected to accumulation gates adjacent to the two sensing dots using the split-gate method [13]. The tank circuit consists of a series NbTiN inductor wirebonded on the PCB, the capacitance between the accumulation gate and the underlying 2DEG, as well as the resistive SET. The path from the 2DEG to the ohmic contact is depleted to minimize resistive leakage to ground. The reflected signal is separated from the incoming carrier with a directional coupler (Mini-Circuits ZEDC-15-2B), amplified at 4 Kelvin (CMT CITLF3) and room temperature before being demodulated and digitized (Keysight M3102A).

Supplementary Note 9. EDSR Driving

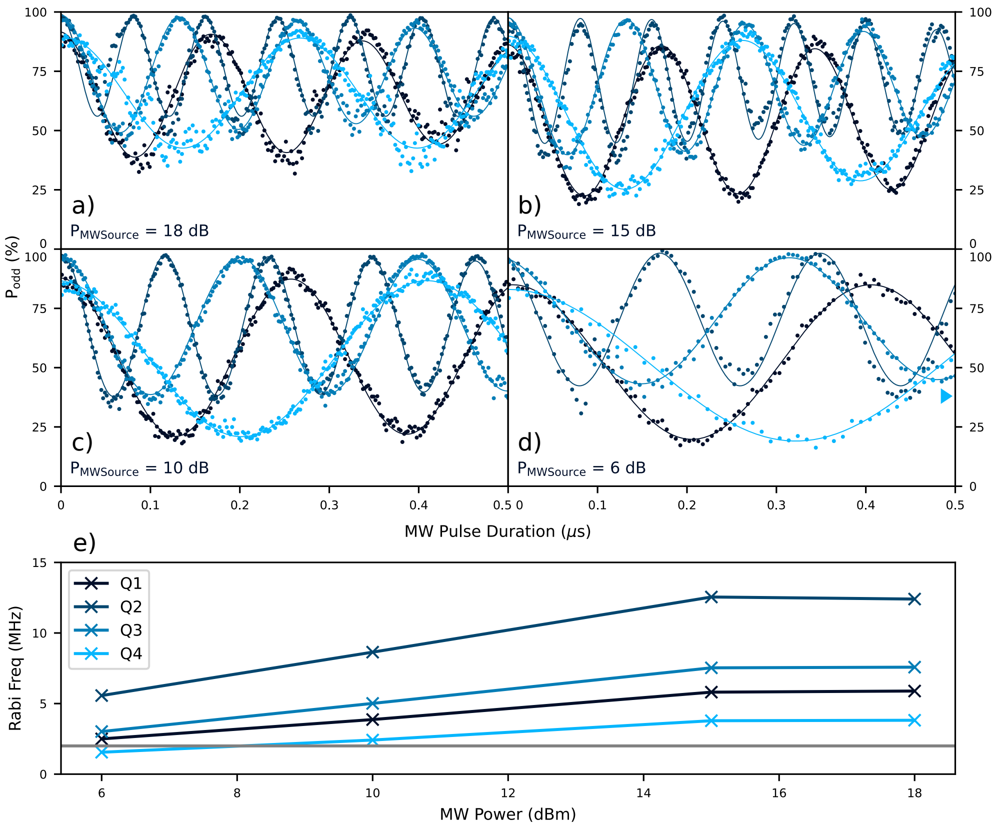

Supplementary Figure 8. **Rabi oscillations at various MW powers** a)-d) Measured (data points) and fitted (solid lines) Rabi oscillations for all four qubits at various powers of the MW-source as annotated in the figures. We point out that the data of Q_4 in panel d) was cropped as indicated by the marker. The fit was performed with the full oscillation. Visibility differences between panel a) and panels b) through d) can be attributed to a retuning of initialization and read out parameters. e) Rabi frequencies plotted against the MW-source powers. Above 15 dBm we observe a saturation on all qubits that is caused by the limitations of the microwave source. For the experiments in the main text we used 6 dBm and adjusted the IQ input amplitudes to achieve a Rabi frequency of around 2 MHz (gray line) on all 4 qubits.

Supplementary Note 10. PIRS Fitting

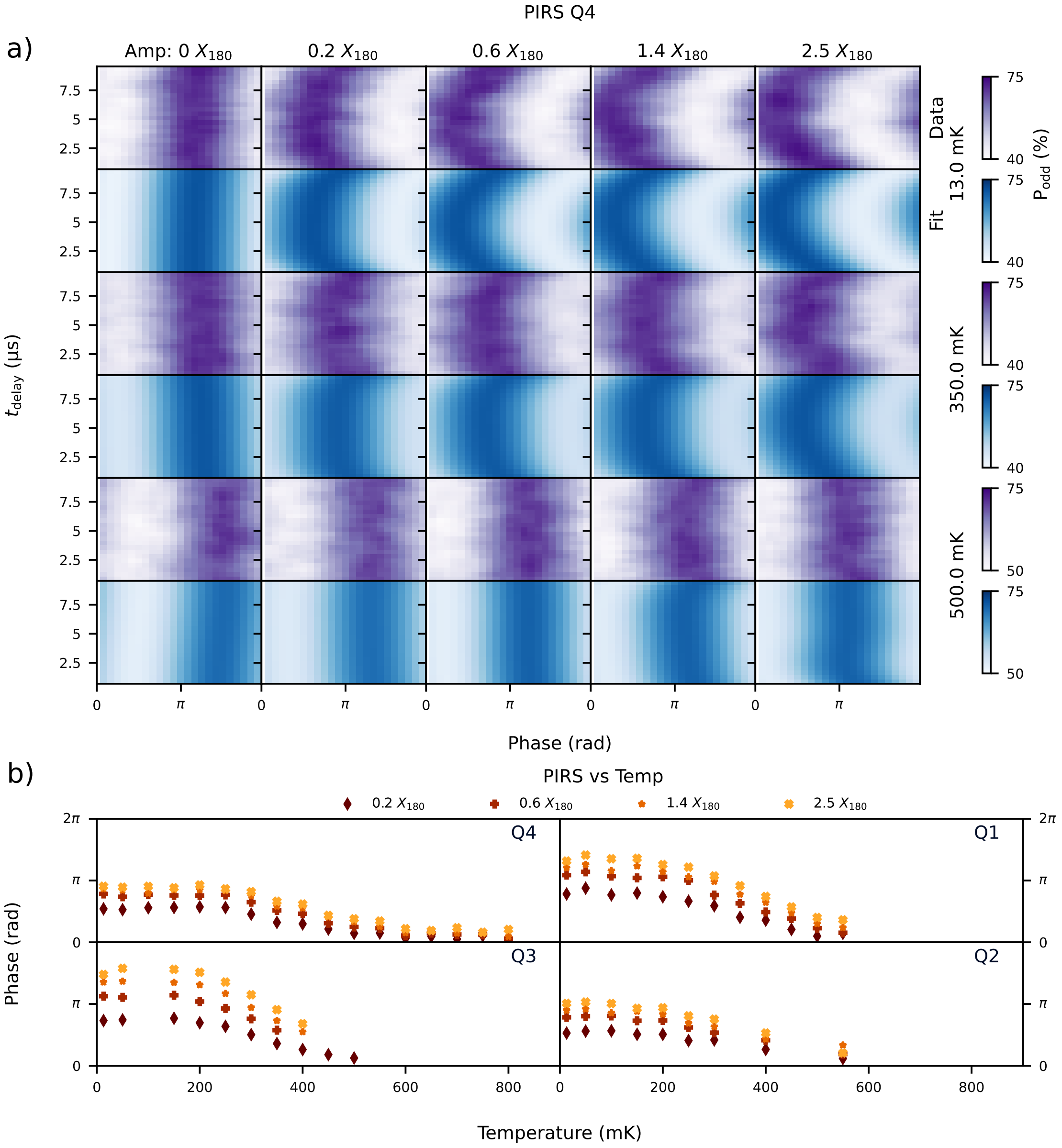

Supplementary Figure 9. **PIRS during EDSR operation** a) Experimental data (purple) and respective fits (blue) for different off-resonant burst amplitudes at three exemplary mixing chamber temperatures for Qubit 4 (see Fig. 2 for the pulse sequence schematic). As the individual traces at constant t_{delay} are both low in visibility due to the long echo pulse sequence used and subject to small signal drifts over the course of the long experiments, we fit the 2D datasets to Supplementary Eq. (24) using a quartic polynomial representation of $\theta_t(t_{\mathrm{delay}}) = \sum_{k=0}^{4} c_k(t_{\mathrm{delay}} - t_0)^k$ to extract the smooth transient phase accumulation from the off-resonant burst as a function of t_{delay}. In the left-most column, the experiment is a standard Hahn-echo sequence, and a constant phase offset is observed at the different temperature settings. We subtract this constant offset from the datasets plotted to the right which are collected at the same temperature in order to isolate the transient effect of the off-resonant burst. b) To quantify the relation between temperature and non-linear transient phase pick-up, we plot the maximum phase accumulation at as a function of mixing chamber temperature for all four qubits. All qubits exhibit the same behaviour whereby the maximum phase pickup is suppressed by operating at higher device temperatures. The positive phase accumulation is consistent with a negative qubit frequency shift given the definition of the rotating frame used in these experiments (see Supplementary Note 4)

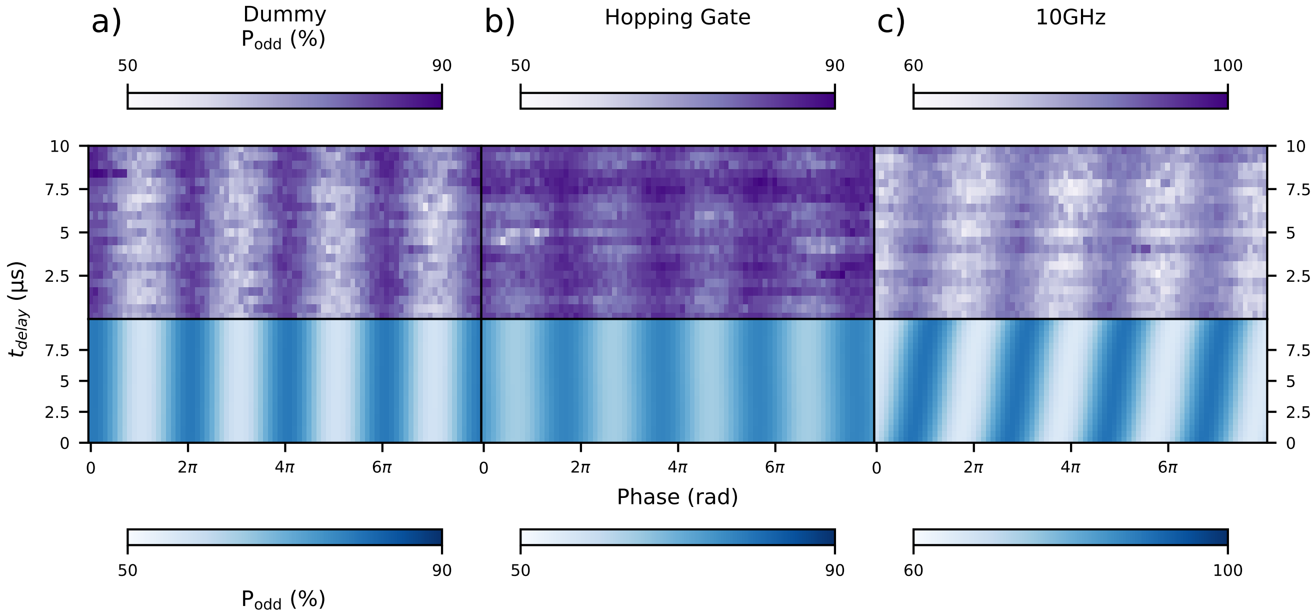

Supplementary Figure 10. **PIRS during baseband operation** a-c) Experimental data (purple) and respective fits (blue) using three variants of the PIRS experiment. a) The Hahn-echo pulse template is implemented on Qubit 1 using hopping gates. The same fitting procedure as described in Supplementary Fig. 9 is used. No off-resonant burst or pulse is applied. b) An X_{180} hopping gate is applied to Qubit 4 in place of the off-resonant burst. Qubit 1 experiences a non-transient phase pickup in addition to a 180 deg phase shift due to the change in measurement parity. The visibility is notably degraded compared to the other experiments due to the effect of baseband pulsing on gate P3. c) A 10 GHz burst is applied with an amplitude and duration energetically commensurate with $0.6X_{180}$ during standard EDSR operation. A transient phase pickup is observed, but no "saturation" effect as in Supplementary Fig. 9 is seen. We believe this is due to the implementation of the decoupling pulse with a hopping gate rather than a resonant gate. The hopping gate itself does not impart a PIRS effect on the device.

Supplementary Note 11. Temperature dependence of spin coherence

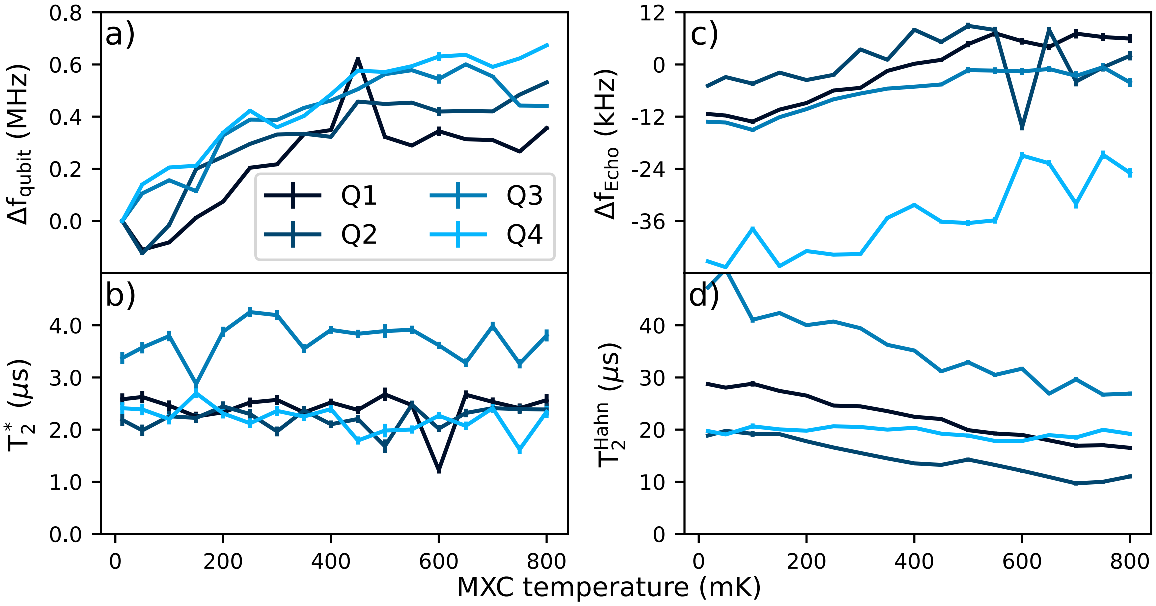

Supplementary Figure 11. **Temperature dependence of T_2^*, T_2^{H} and qubit frequencies** a-b) Data extracted from Ramsey experiments (approx. 30 min) conducted using EDSR control as a function of mixing chamber temperature. The measured oscillations can be fit to extract (a) the qubit frequency and (b) the Ramsey decay time T_2^* per Supplementary Note 4. Discounting spurious jumps in qubit frequency on the order of 100 kHz which we attribute to slow charge fluctuators, all qubit frequencies increase monotonically as a function of temperature within the range we are able to measure. The Ramsey decay times are notably unaffected by the device temperature for all four qubits. c-d) Hahn-echo experiments (approx. 30 min) conducted using EDSR control as a function of mixing chamber temperature. The measured oscillations can be fit to extract (c) the difference in qubit frequency before and after the decoupling pulse and (d) the Hahn-echo decay time T_2^{H} per Supplementary Note 4. The systematic difference in qubit frequencies is predominantly attributed to the PIRS induced by the decoupling X_{180} pulse as it has a greater duration than the initial X_{90} pulse. This systematic frequency difference appears to converge to zero as the mixing chamber temperature is increases, which is consistent with the observation that an increased device temperature mitigates the PIRS effect (see Supplementary Fig. 9). Hahn-echo decay times for all qubits except Qubit 4 monotonically decrease as a function of mixing chamber temperature. Qubit 4's coherence remains constant with the changing temperature. The plotted error bars in all panels represent the standard deviation of the fitted parameters.

262 **Supplementary Note 12. Lever arm, tunnel coupling, and electron temperature**

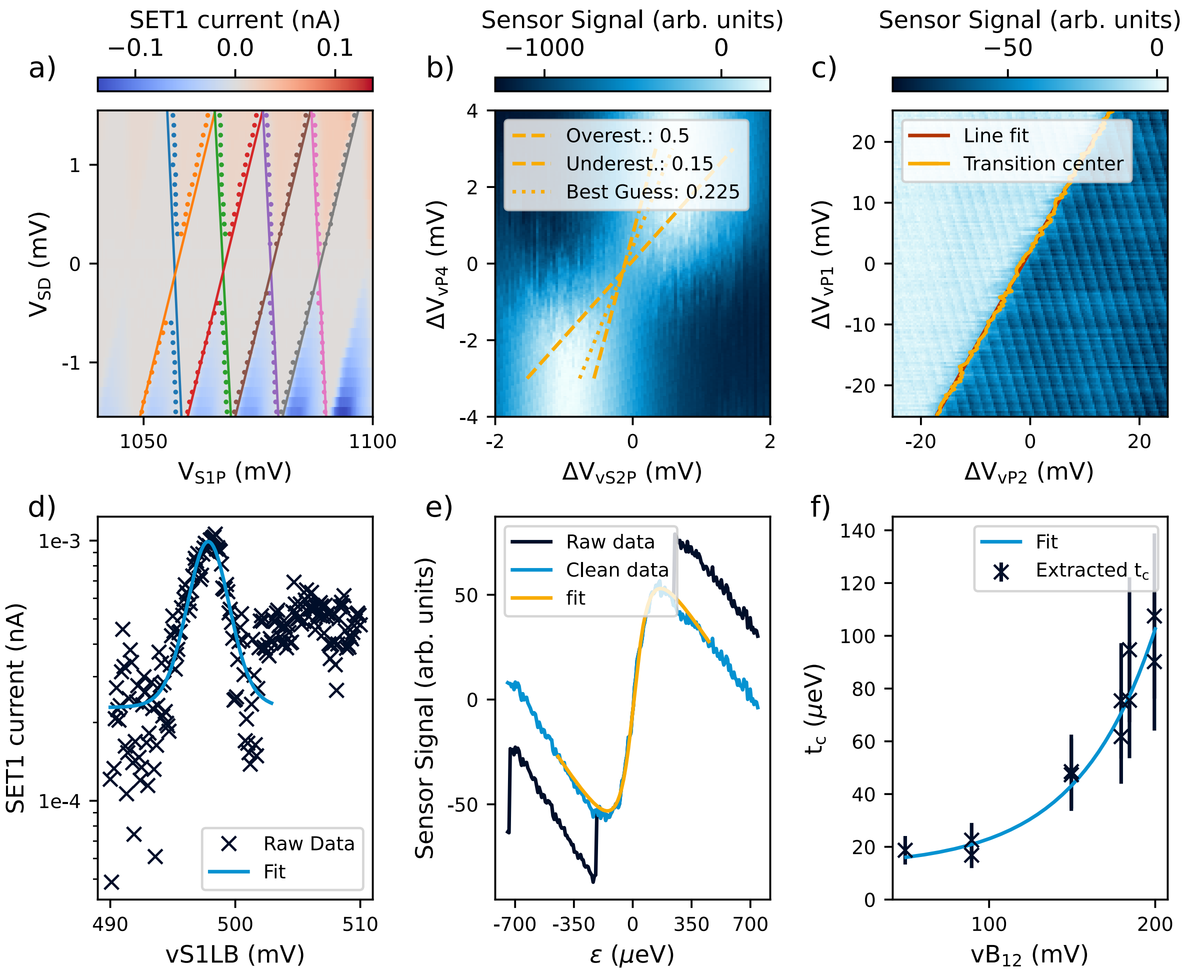

Supplementary Figure 12. **Lever arm, electron temperature and tunnel coupling measurements** a) Coulomb diamonds of SET 1 showing the extracted slopes used to calculate the lever arm of the sensing dot plunger. b) An exemplaray interdot transition of sensing dot 2 and quantum dot 4. The shift of the coulomb peak (bright white line) due to the added charge on quantum do D_4 is used for determining the lever arm ratio between the sensing dot plunger and the quantum dot plunger. Dashed and dotted lines show the manual over- and under-estimation as well as the best fit. The latter takes into account that both lever arms for vS1P and vS2P have to be in an agreement when propagated through the array. c) An exemplary interdot transition between quantum dots 1 and 2 used for determining the lever arm ratio between the two plunger gates. We fit the interdot transition line-by-line to extract a consistent center point followed by a linear fit through all center points to determine the slope. d) Coulomb peak and fit to extract a bound on the electron temperature of about $T_e < 175\,\mathrm{mK}$. e) Exemplary fit of an interdot transition to extract the tunnel coupling [14]. The detuning lever arm as computed in panels a-c) as well as the electron temperature from d) are used in this fitting procedure. The sensing dot plunger is virtualized with respect to the detuning, and discrete steps in the measured signal due to finite AWG resolution are extracted from the measured transition prior to fitting. f) Extracted tunnel couplings between dots 1 and 2 plotted against the respective barrier voltage showing the expected exponential dependency. The plotted error bars represent the standard deviation of the fit using the nominal value of the fitted lever arm and electron temperature.

Supplementary Table II. Lever arms in the 2x2 array. Virtualized sensor dot plungers (vS1P and vS2P) and virtualized loading barriers (vS1LB and vS2LB) are extracted directly via Coulomb diamonds. Virtualized quantum dot plungers (vP1 - vP4) are inferred by propagating the slope of interdot transitions through the array. The uncertainty of the virtual plunger gates is dominated by the uncertainty of propagating the sensor lever arm into the 2x2 array.

Gate	Lever arm
vS1P	0.1542 ± 0.0017
vS1LB	0.0155 ± 0.0004
vS2P	0.1537 ± 0.0029
vS2LB	0.0151 ± 0.0002
vP1	0.0289 ± 0.0083
vP2	0.0455 ± 0.0131
vP3	0.0290 ± 0.0084
vP4	0.0349 ± 0.0101

[1] M. Pioro-Ladrière, T. Obata, Y. Tokura, Y.-S. Shin, T. Kubo, K. Yoshida, T. Taniyama, and S. Tarucha, Electrically driven single-electron spin resonance in a slanting zeeman field, Nature Physics **4**, 776–779 (2008).

[2] J. Yoneda, K. Takeda, T. Otsuka, T. Nakajima, M. R. Delbecq, G. Allison, T. Honda, T. Kodera, S. Oda, Y. Hoshi, N. Usami, K. M. Itoh, and S. Tarucha, A quantum-dot spin qubit with coherence limited by charge noise and fidelity higher than 99.9%, Nature Nanotechnology **13**, 102–106 (2017).

[3] T. Tanttu, B. Hensen, K. W. Chan, C. H. Yang, W. W. Huang, M. Fogarty, F. Hudson, K. Itoh, D. Culcer, A. Laucht, A. Morello, and A. Dzurak, Controlling spin-orbit interactions in silicon quantum dots using magnetic field direction, Physical Review X **9**, 10.1103/physrevx.9.021028 (2019).

[4] M. Ortner and L. G. Coliado Bandeira, Magpylib: A free python package for magnetic field computation, SoftwareX **11**, 100466 (2020).

[5] M. Aldeghi, R. Allenspach, A. Vervelaki, D. Jetter, K. Bagani, F. Braakman, M. Poggio, and G. Salis, Simulation and measurement of stray fields for the manipulation of spin-qubits in one- and two-dimensional arrays (2024), arXiv:2410.08865 [cond-mat.mes-hall].

[6] S. G. J. Philips, M. T. Madzik, S. V. Amitonov, S. L. de Snoo, M. Russ, N. Kalhor, C. Volk, W. I. L. Lawrie, D. Brousse, L. Tryputen, B. P. Wuetz, A. Sammak, M. Veldhorst, G. Scappucci, and L. M. K. Vandersypen, Universal control of a six-qubit quantum processor in silicon, Nature **609**, 919–924 (2022).

[7] M. J. Donahue and D. G. Porter, *OOMMF user's guide, version 1.0* (National Institute of Standards and Technology, Gaithersburg, MD, 1999).

[8] M. Aldeghi, R. Allenspach, and G. Salis, Modular nanomagnet design for spin qubits confined in a linear chain, Applied Physics Letters **122**, 10.1063/5.0139670 (2023).

[9] R. Hanson, L. P. Kouwenhoven, J. R. Petta, S. Tarucha, and L. M. K. Vandersypen, Spins in few-electron quantum dots, Reviews of Modern Physics **79**, 1217–1265 (2007).

[10] M. A. Nielsen, A simple formula for the average gate fidelity of a quantum dynamical operation, Physics Letters A **303**, 249 (2002).

[11] F. van Riggelen-Doelman, C.-A. Wang, S. L. de Snoo, W. I. L. Lawrie, N. W. Hendrickx, M. Rimbach-Russ, A. Sammak, G. Scappucci, C. Déprez, and M. Veldhorst, Coherent spin qubit shuttling through germanium quantum dots, Nature Communications **15**, 10.1038/s41467-024-49358-y (2024).

[12] C.-A. Wang, V. John, H. Tidjani, C. X. Yu, A. S. Ivlev, C. Déprez, F. van Riggelen-Doelman, B. D. Woods, N. W. Hendrickx, W. I. L. Lawrie, L. E. A. Stehouwer, S. D. Oosterhout, A. Sammak, M. Friesen, G. Scappucci, S. L. de Snoo, M. Rimbach-Russ, F. Borsoi, and M. Veldhorst, Operating semiconductor quantum processors with hopping spins, Science **385**, 447–452 (2024).

[13] Y.-Y. Liu, S. Philips, L. Orona, N. Samkharadze, T. McJunkin, E. MacQuarrie, M. Eriksson, L. Vandersypen, and A. Yacoby, Radio-frequency reflectometry in silicon-based quantum dots, Physical Review Applied **16**, 10.1103/physrevapplied.16.014057 (2021).

[14] L. DiCarlo, H. J. Lynch, A. C. Johnson, L. I. Childress, K. Crockett, C. M. Marcus, M. P. Hanson, and A. C. Gossard, Differential charge sensing and charge delocalization in a tunable double quantum dot, Phys. Rev. Lett. **92**, 226801 (2004).
